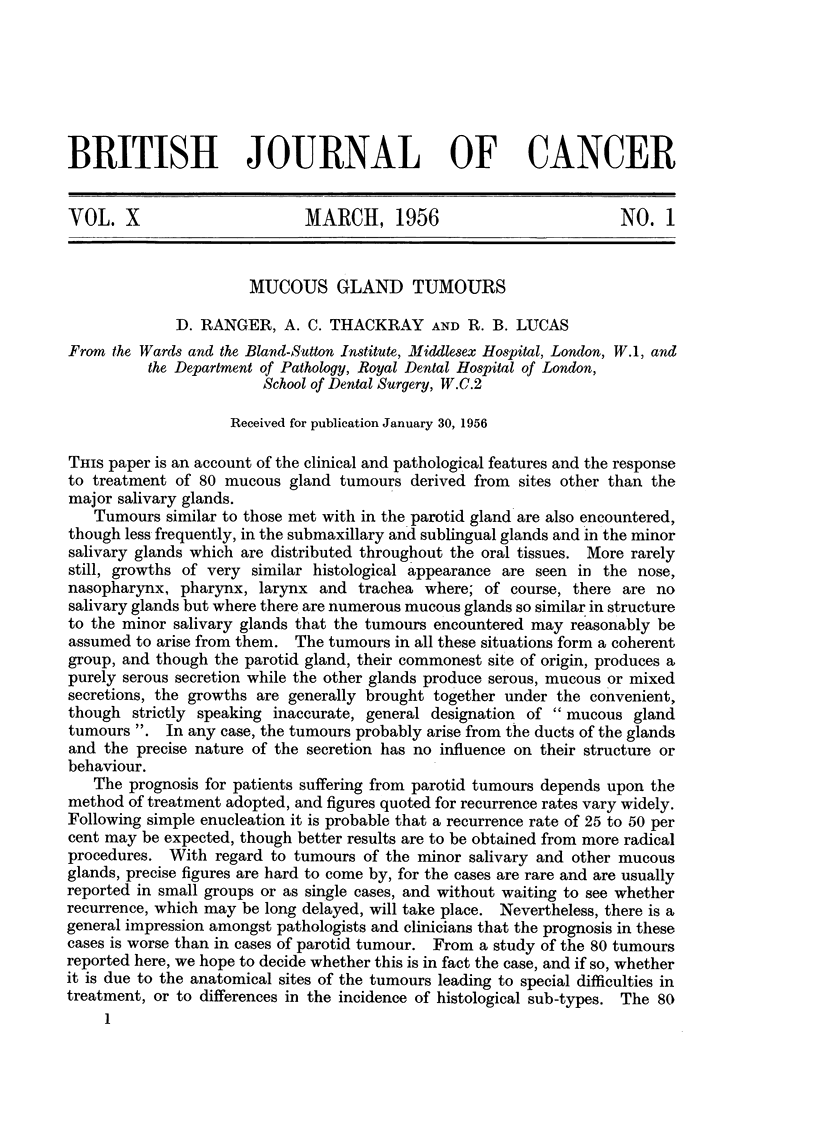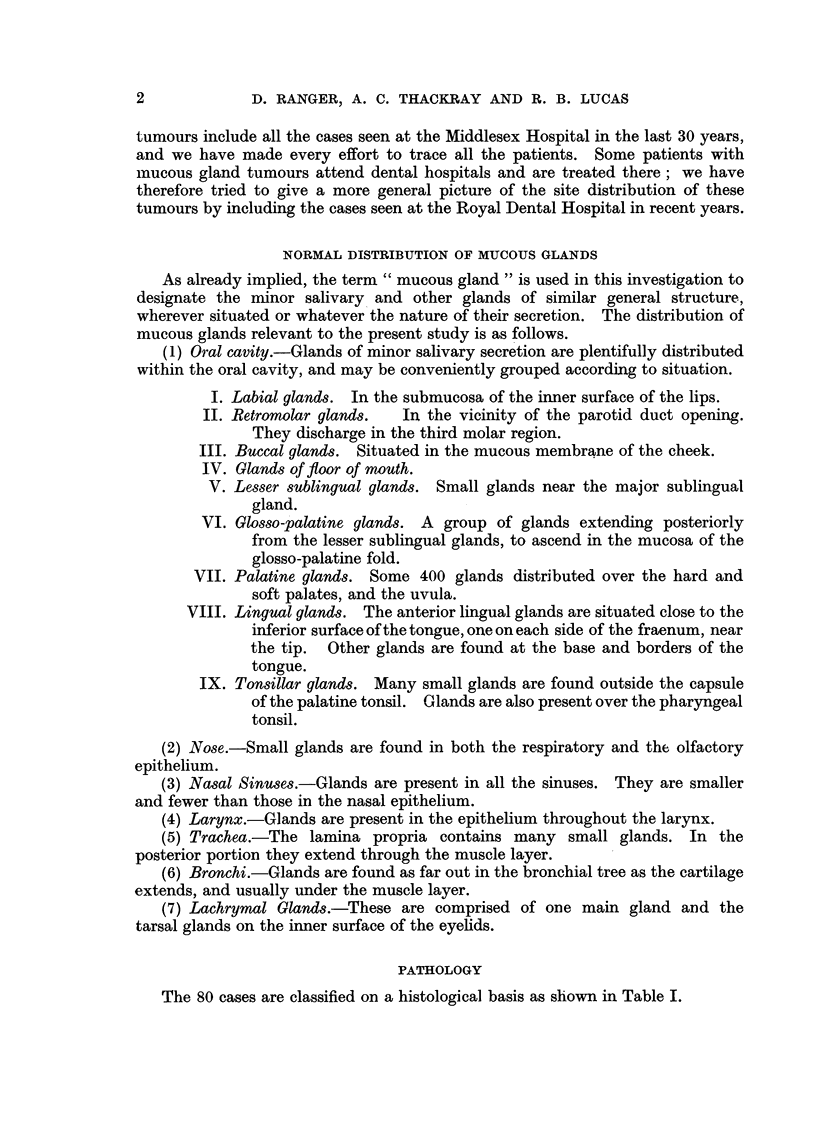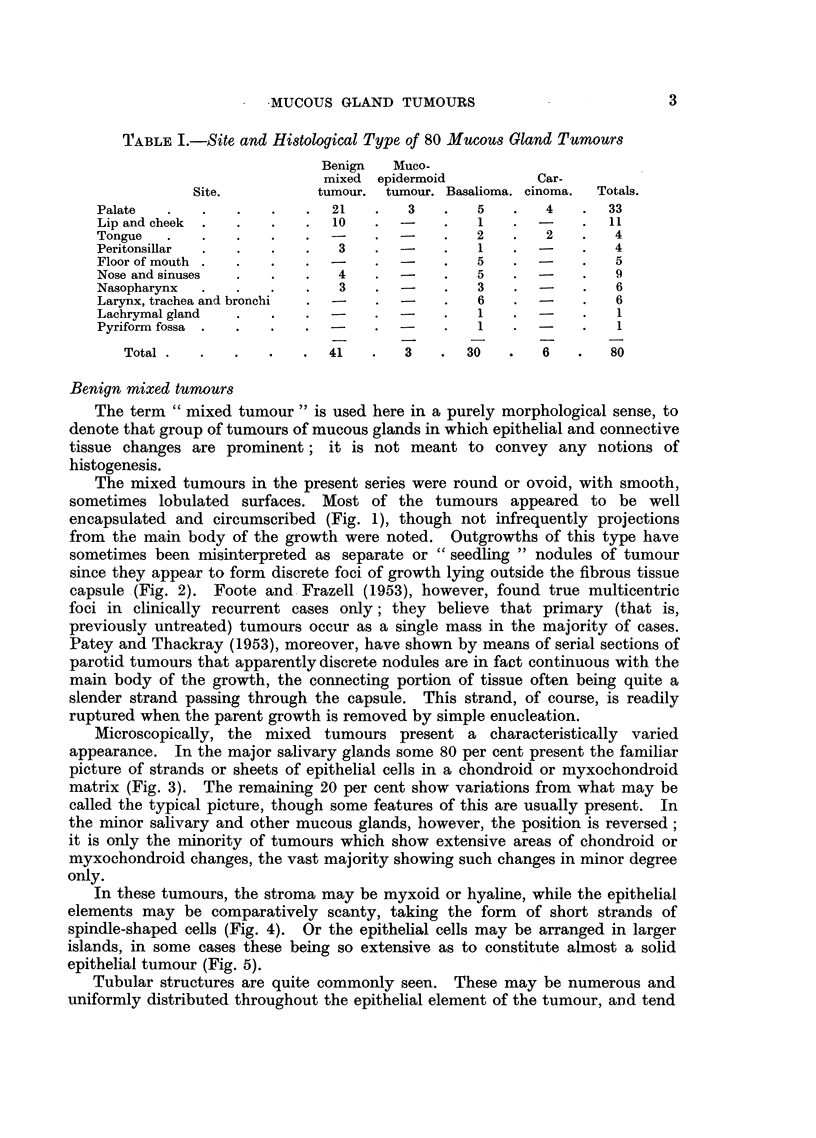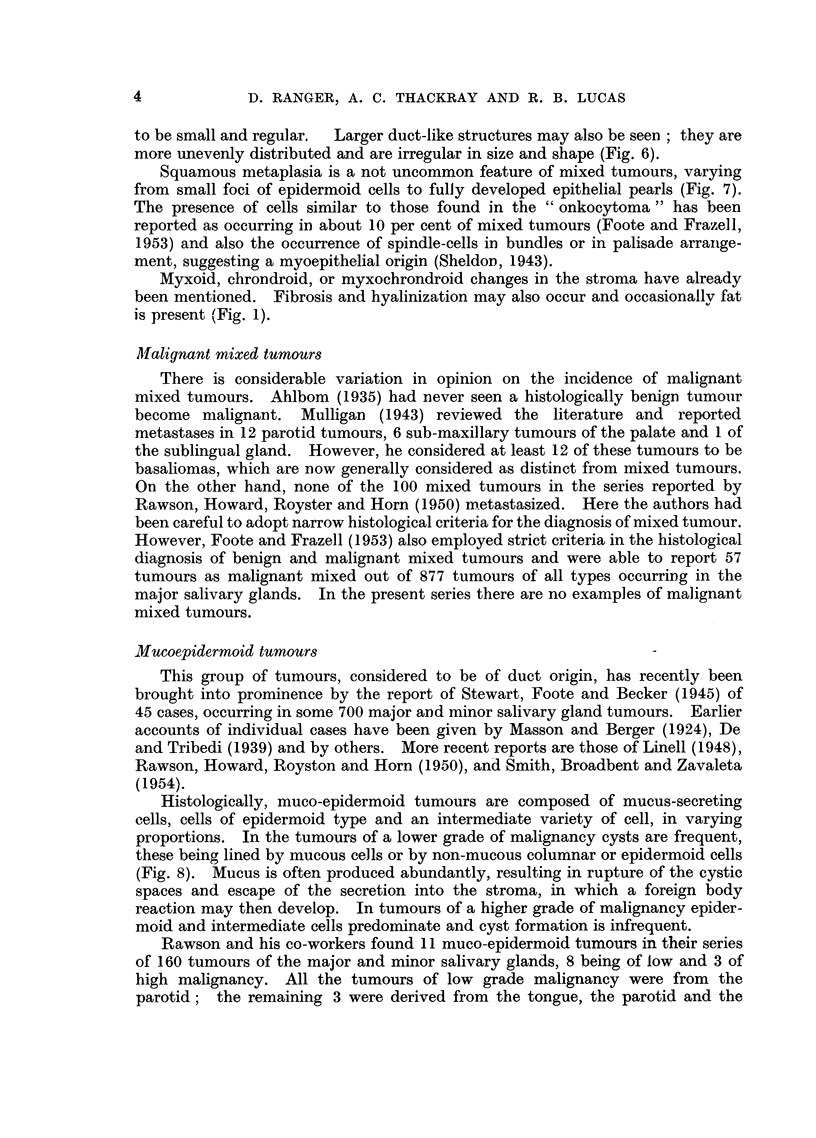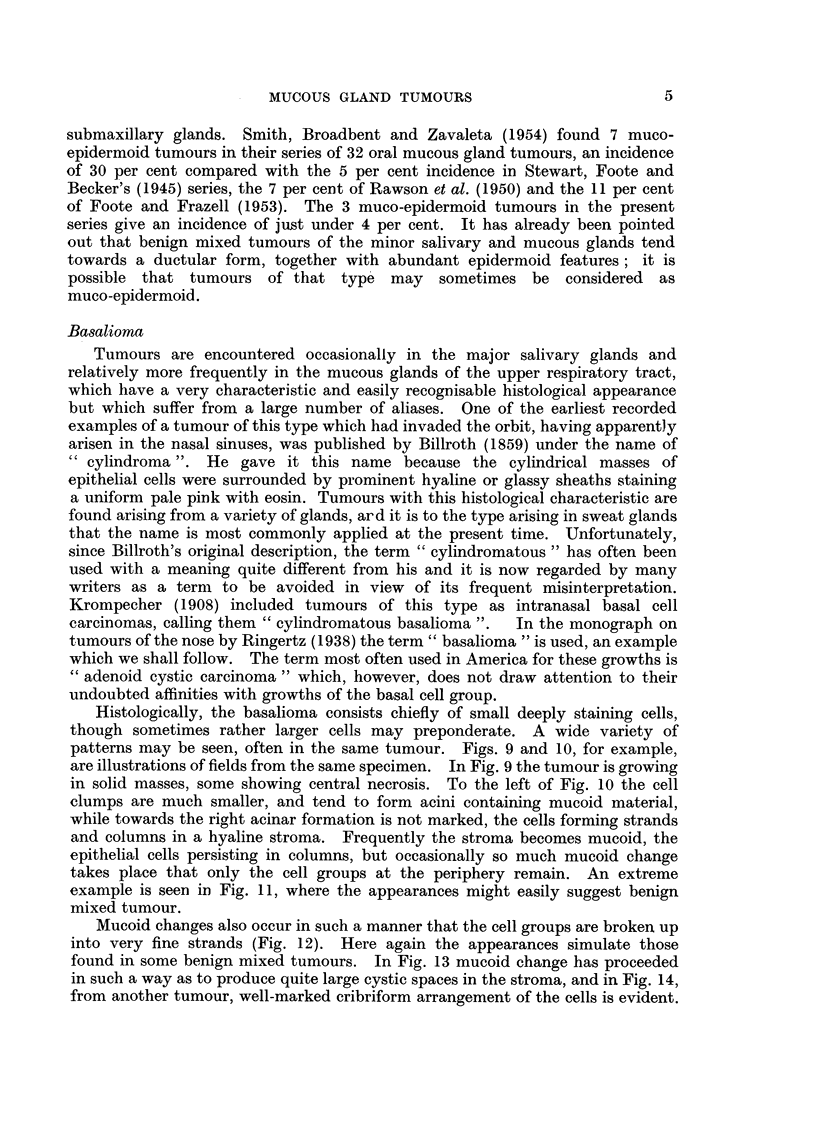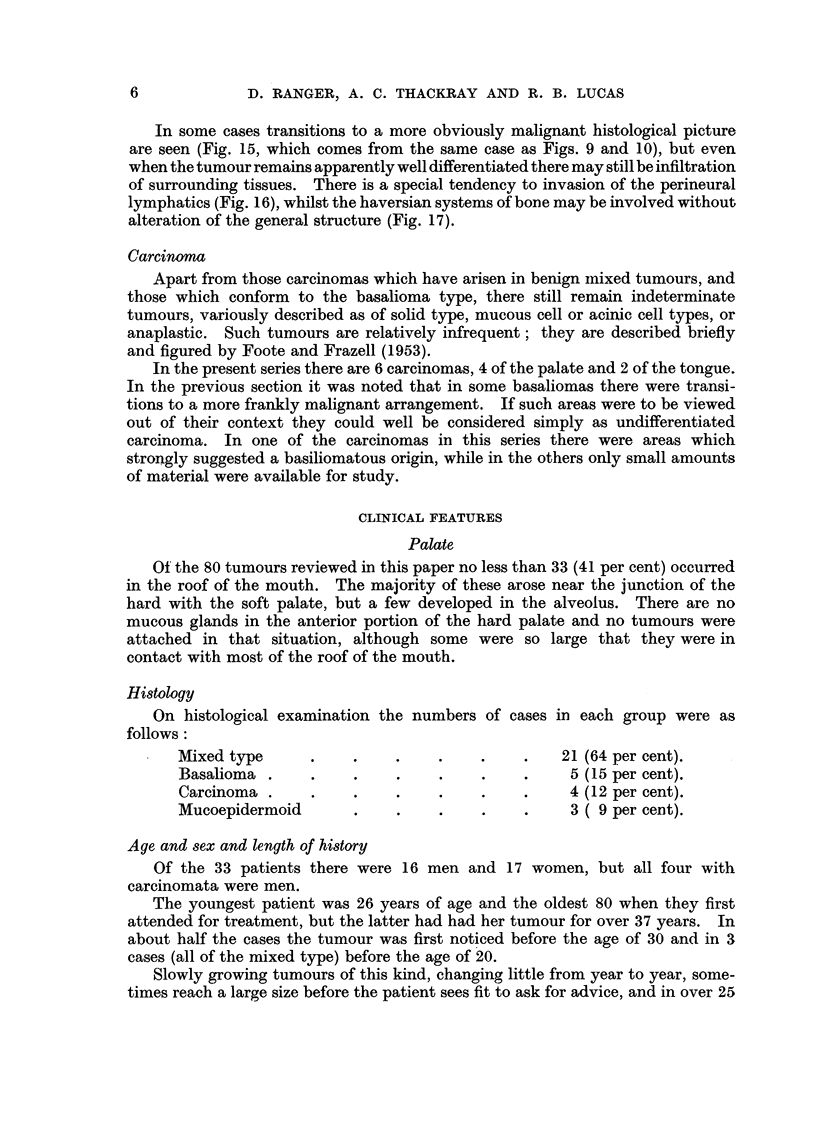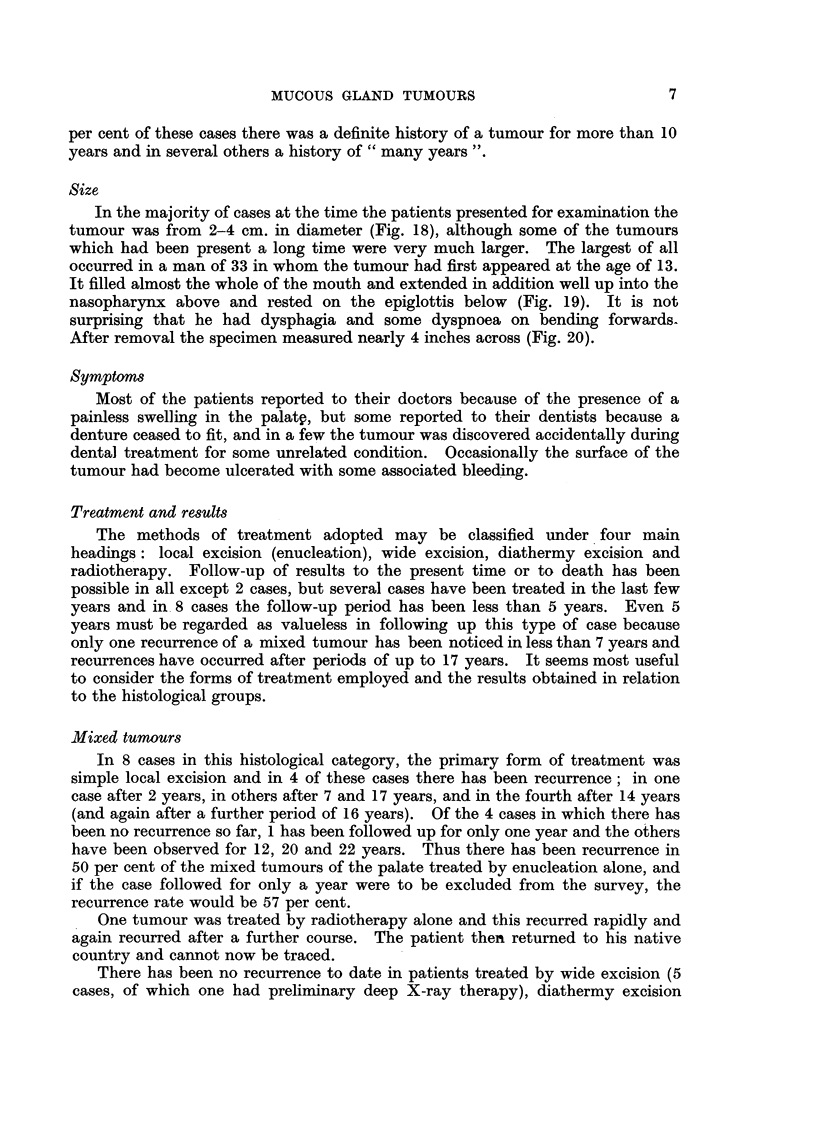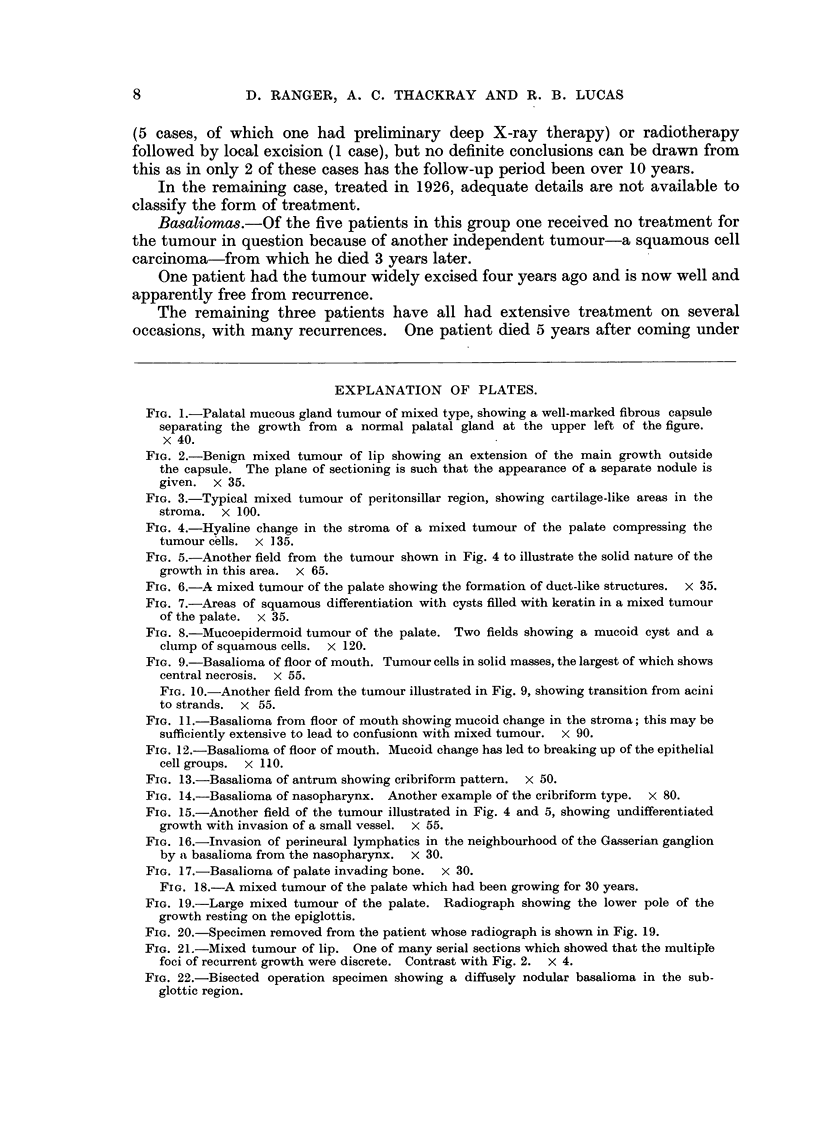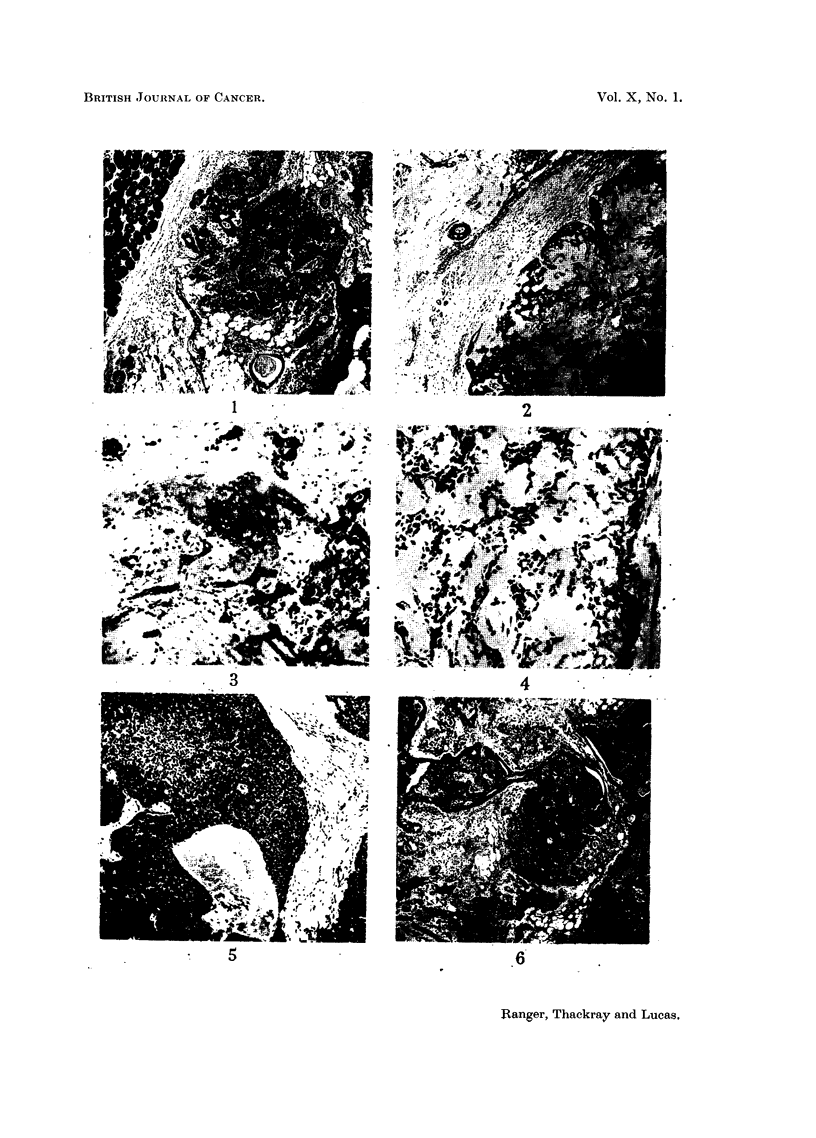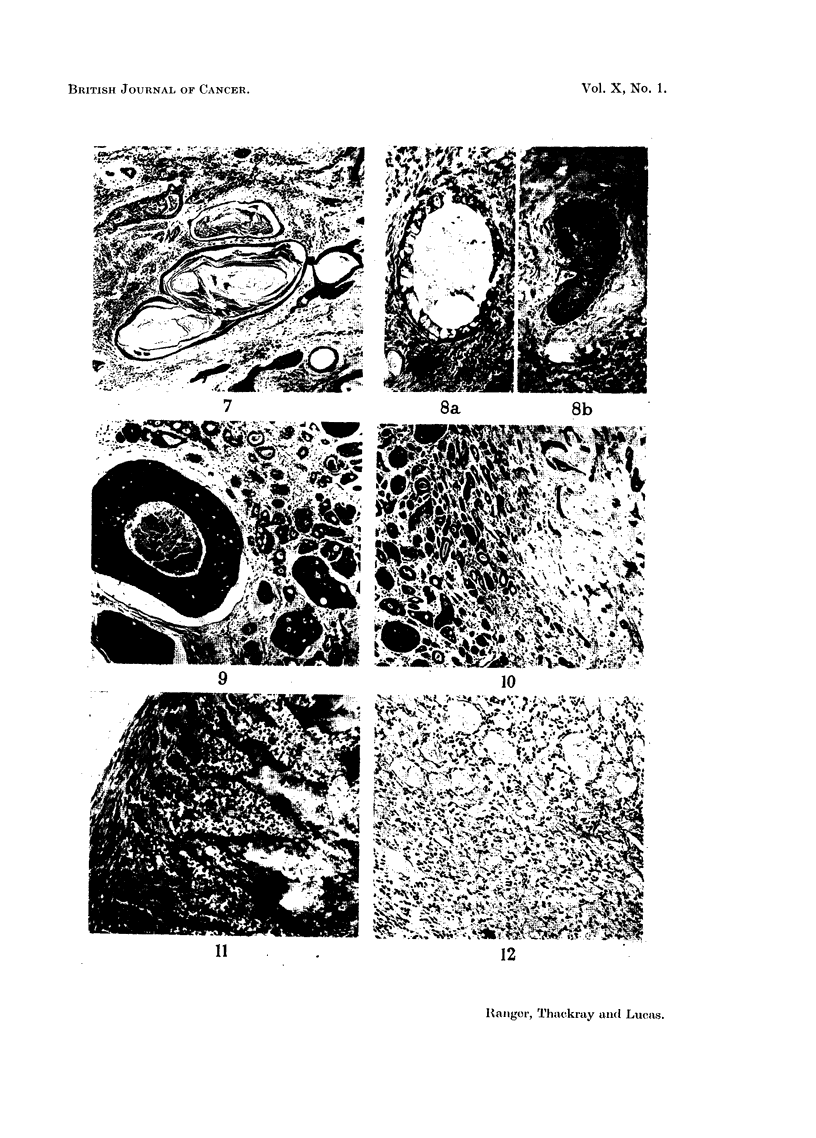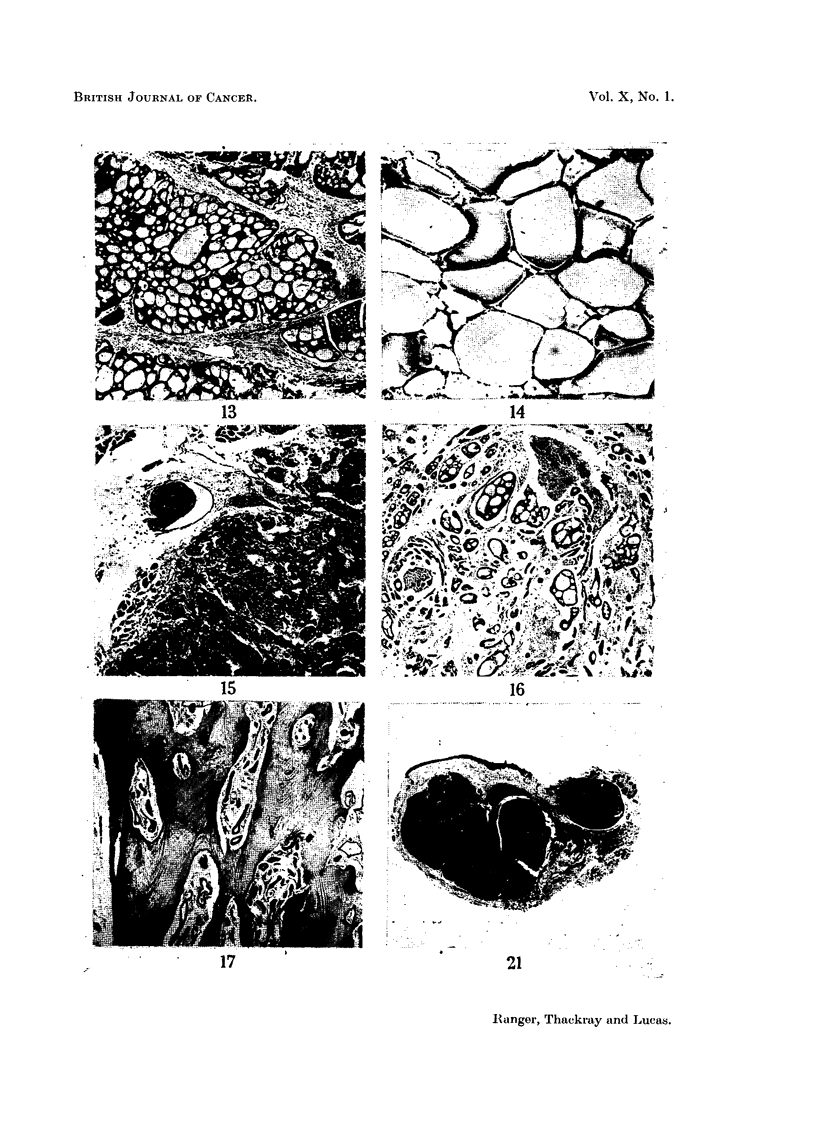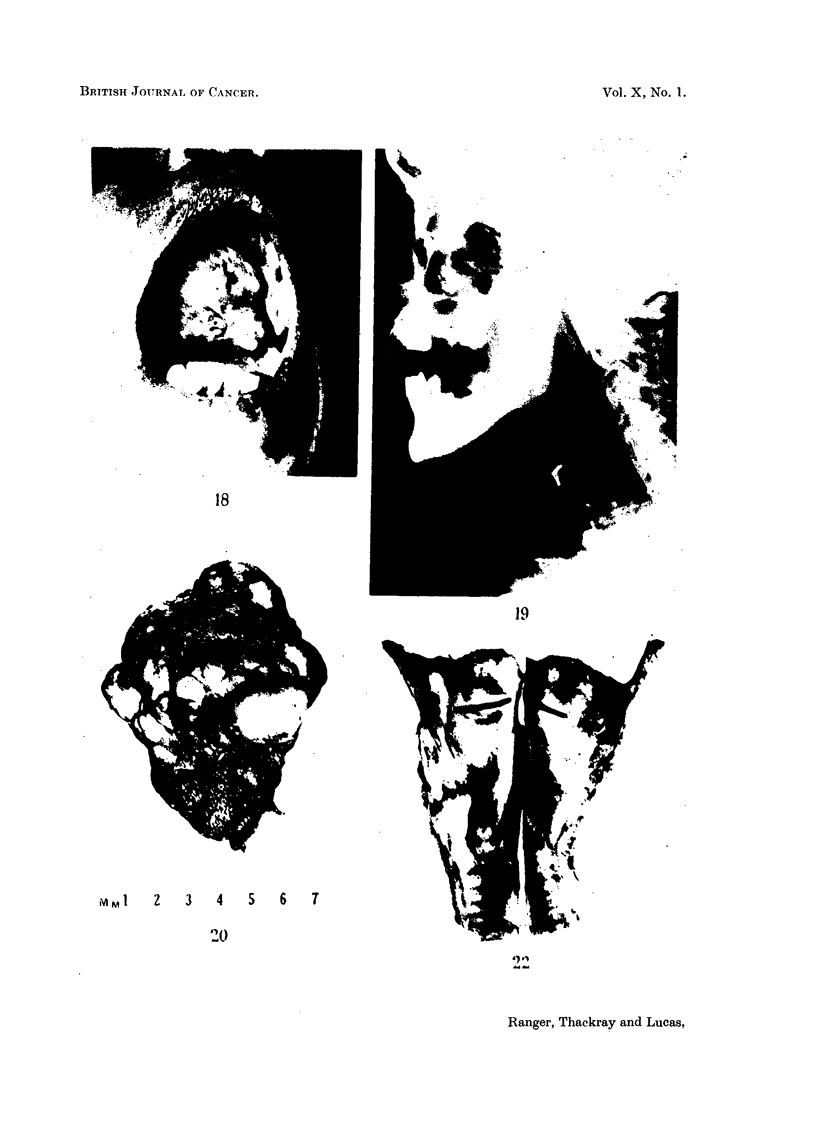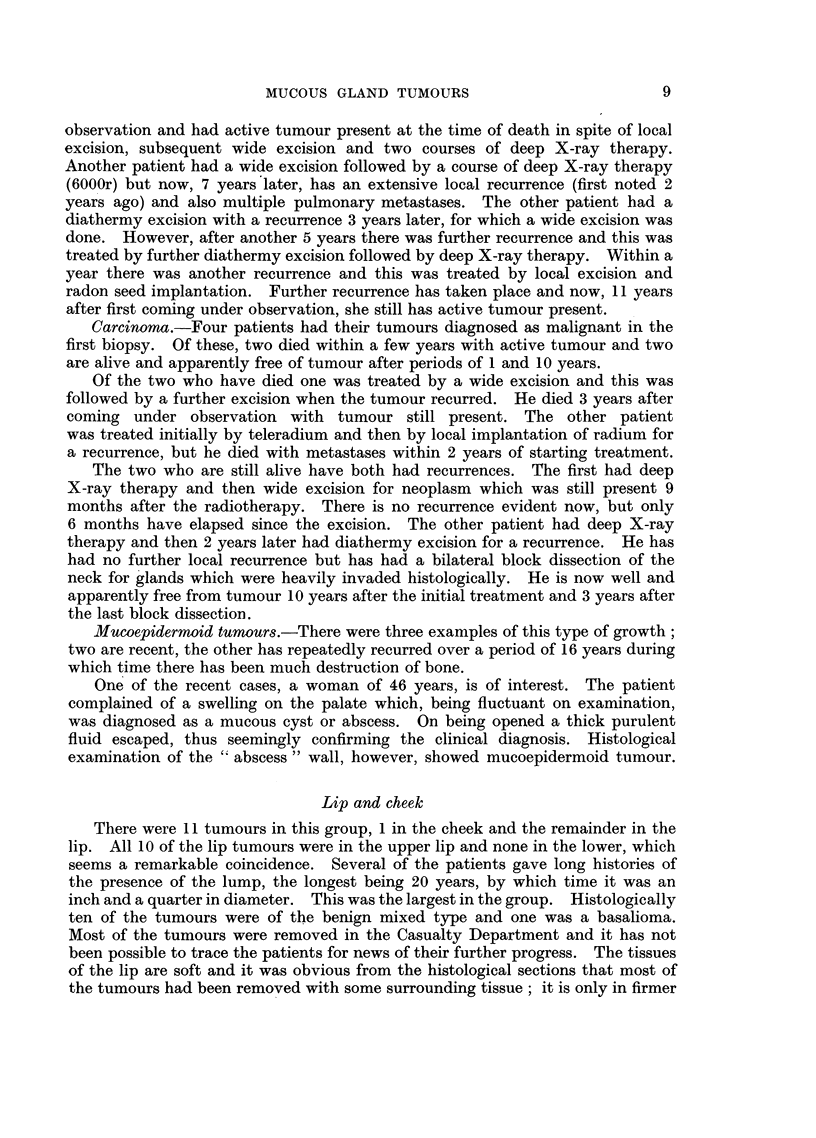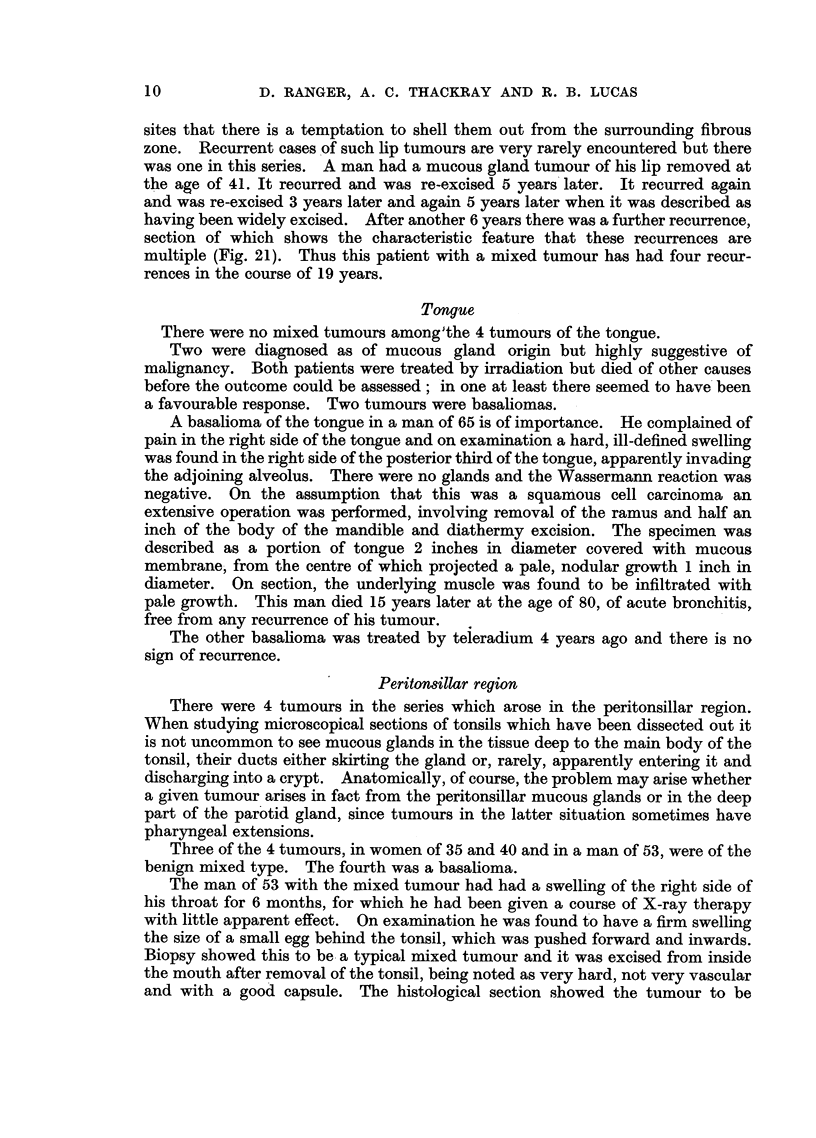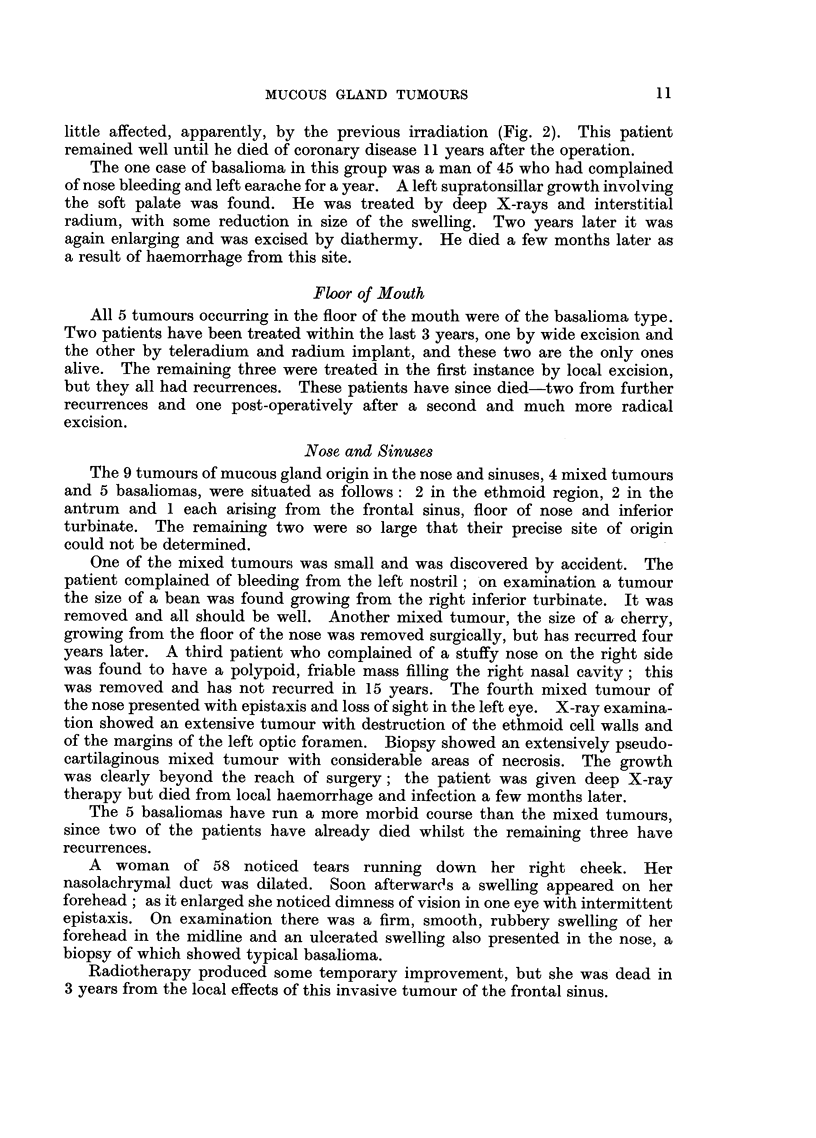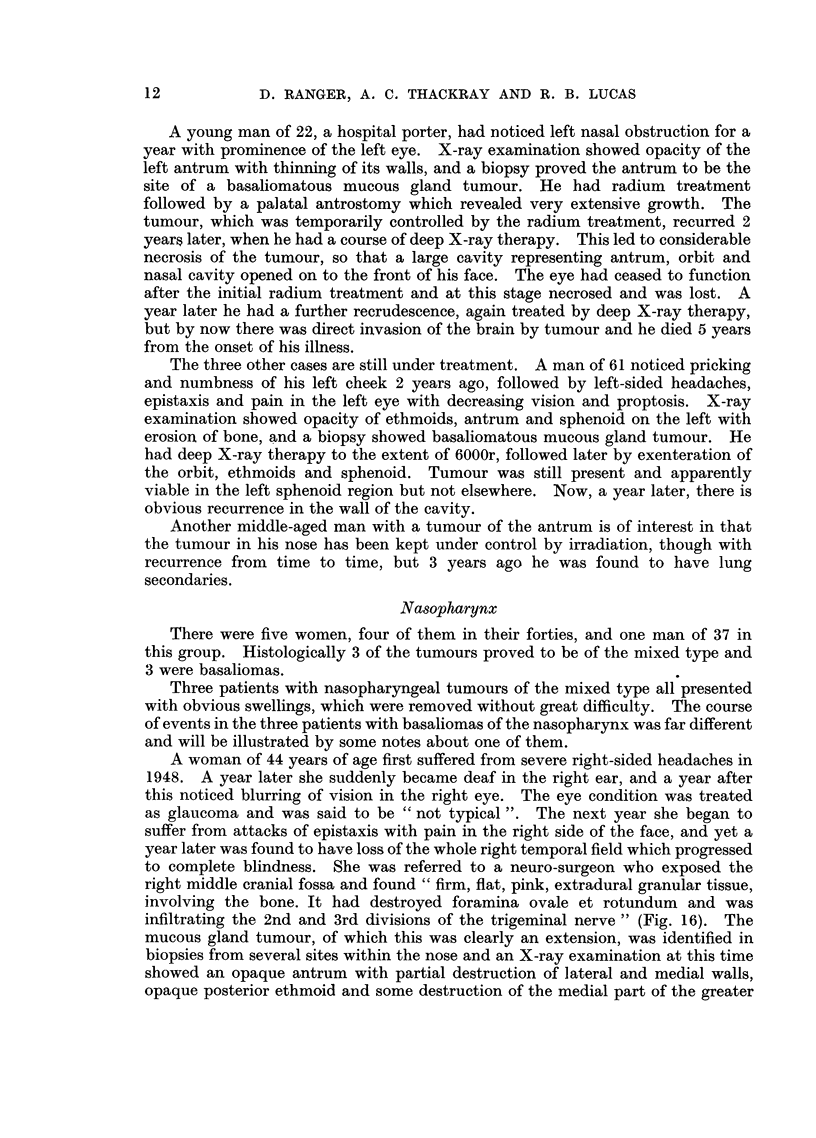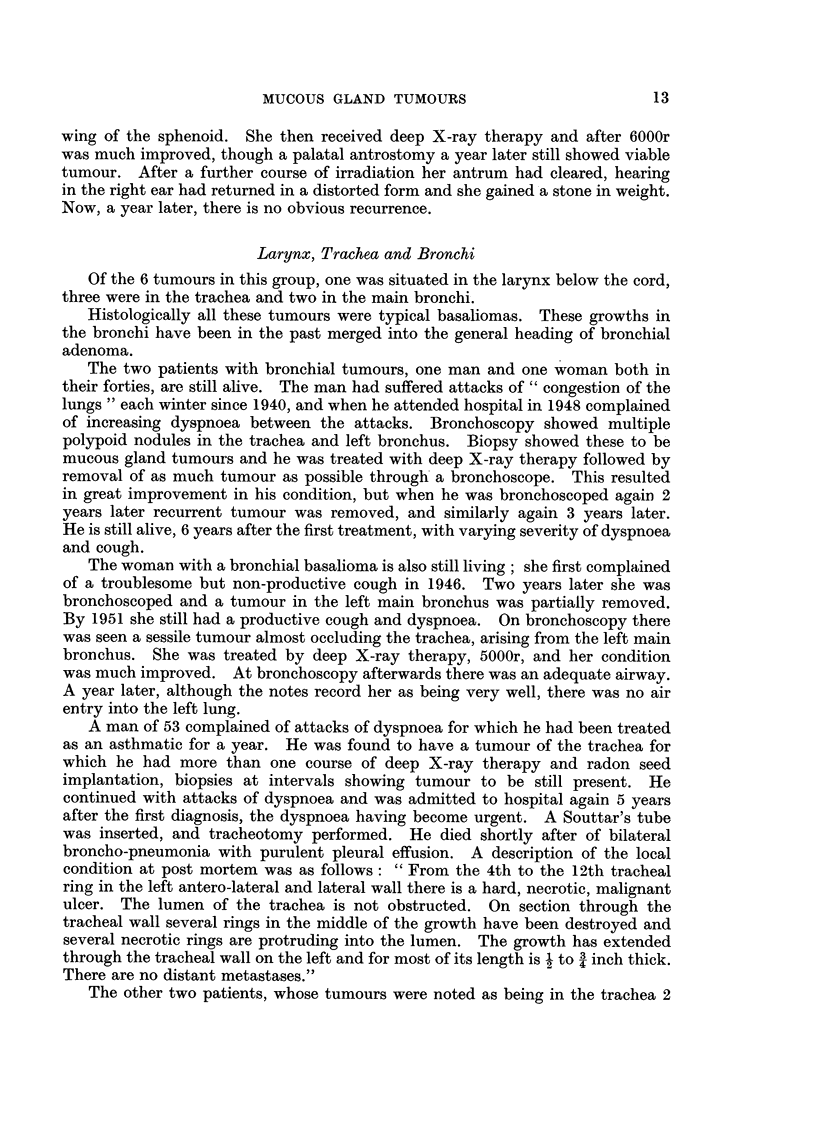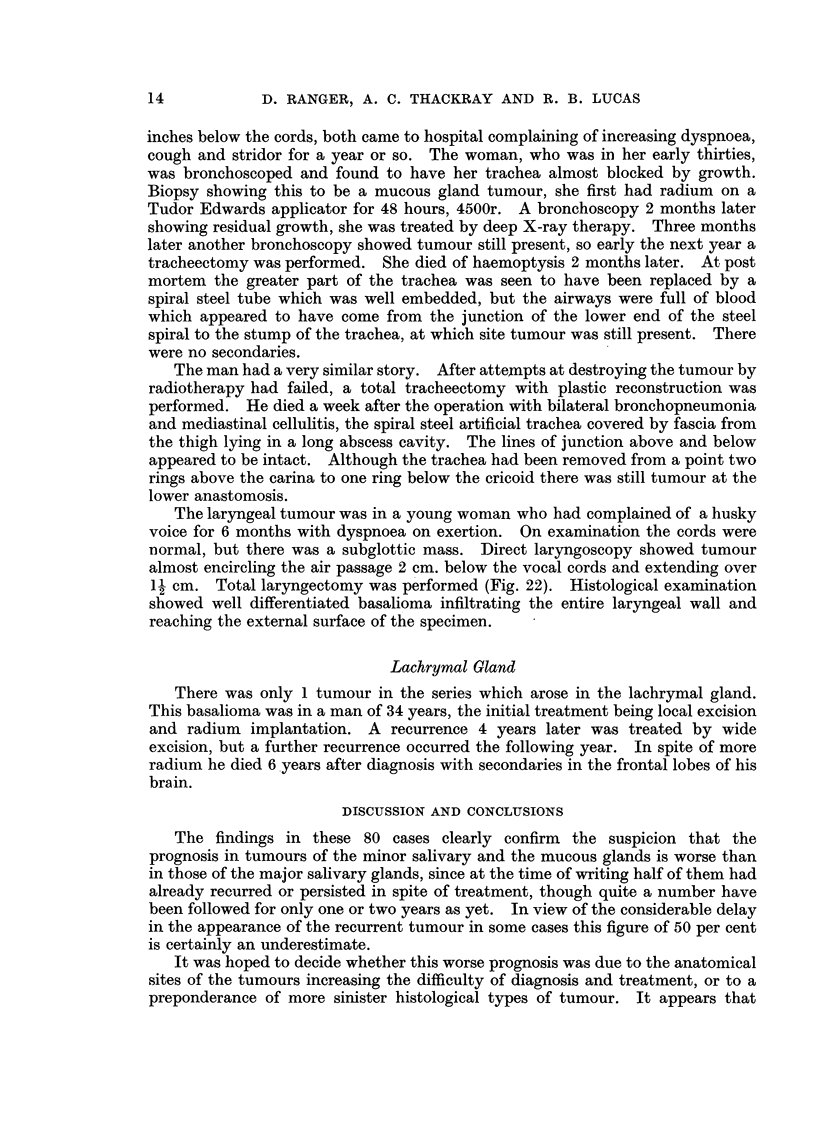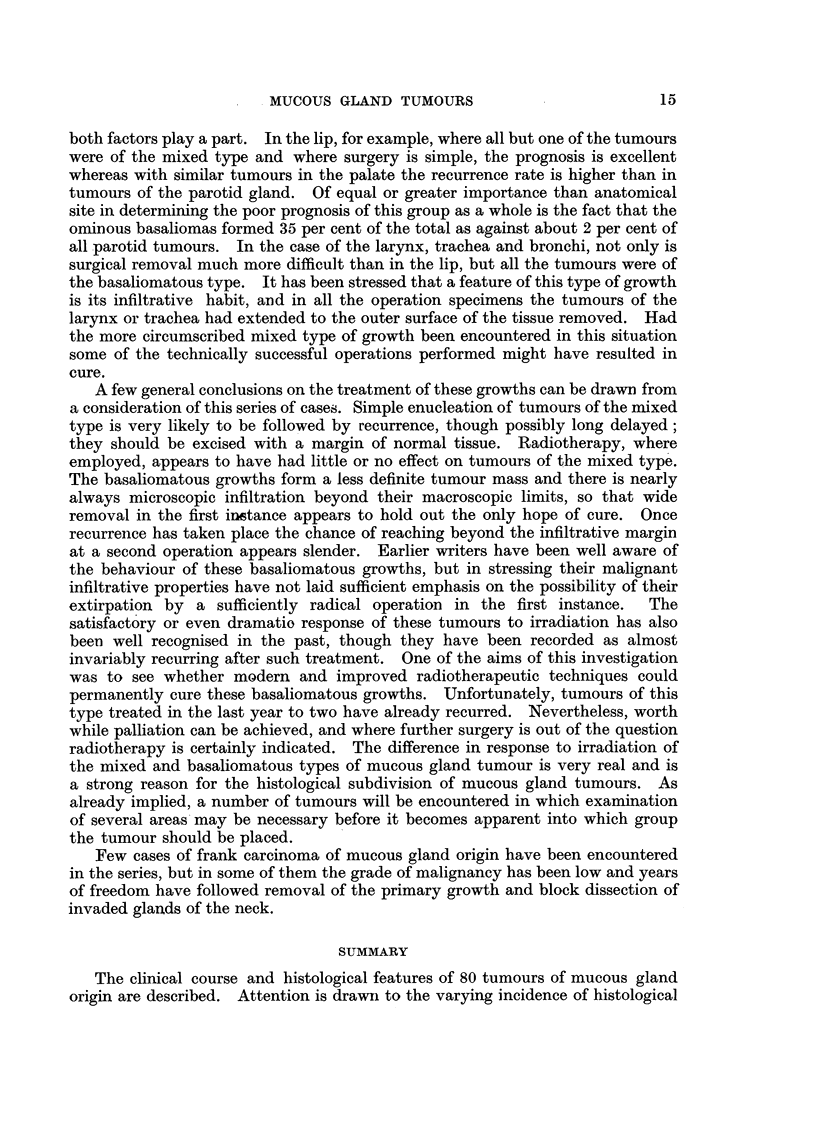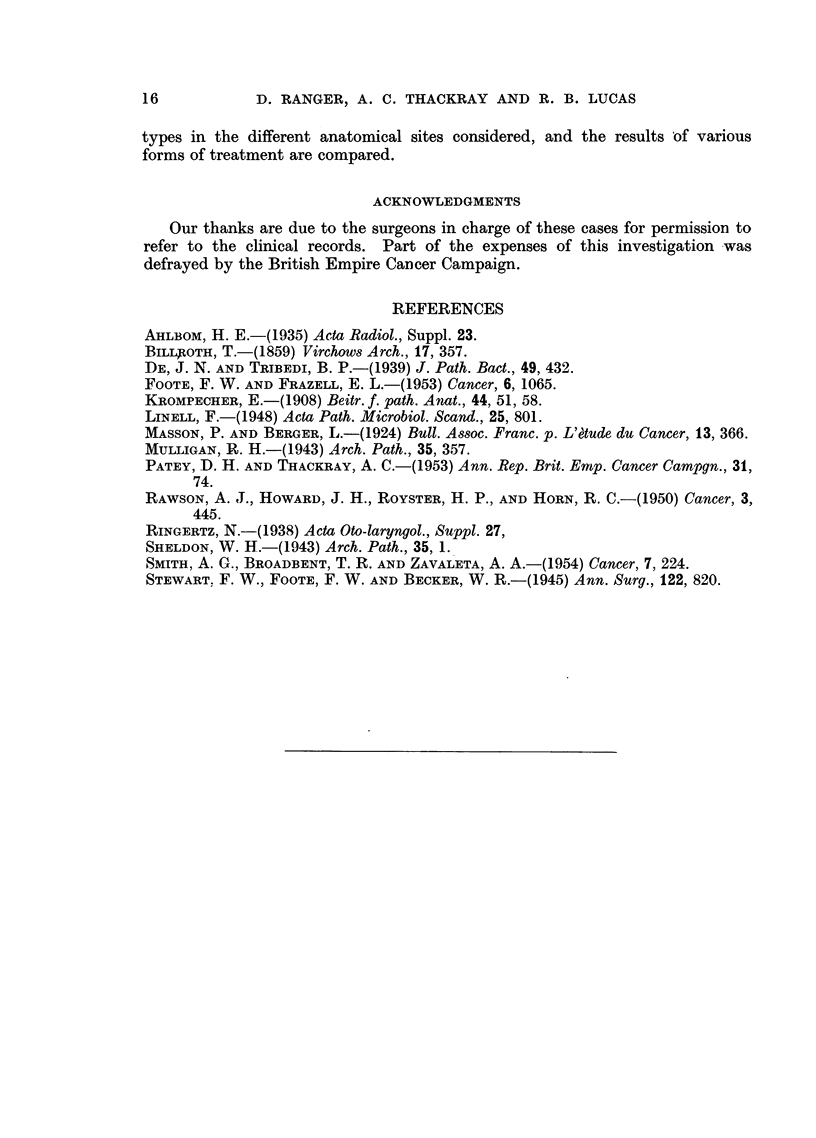# Mucous Gland Tumours

**DOI:** 10.1038/bjc.1956.1

**Published:** 1956-03

**Authors:** D. Ranger, A. C. Thackray, R. B. Lucas

## Abstract

**Images:**


### BRITISH JOURNAL OF CANCER

VOL. X MARCH, 1956 NO. 1

### MUCOUS GLAND TUMOURS

D. RANGER, A. C. THACKRAY AND R. B. LUCAS

*From the Wards and the Bland-Sutton Institute, Middlesex Hospital, London, W.1, and the Department of Pathology, Royal Dental Hospital of London, School of Dental Surgery, W.C.2*

Received for publication January 30, 1956

THIS paper is an account of the clinical and pathological features and the response to treatment of 80 mucous gland tumours derived from sites other than the major salivary glands.

Tumours similar to those met with in the parotid gland are also encountered, though less frequently, in the submaxillary and sublingual glands and in the minor salivary glands which are distributed throughout the oral tissues. More rarely still, growths of very similar histological appearance are seen in the nose, nasopharynx, pharynx, larynx and trachea where; of course, there are no salivary glands but where there are numerous mucous glands so similar in structure to the minor salivary glands that the tumours encountered may reasonably be assumed to arise from them. The tumours in all these situations form a coherent group, and though the parotid gland, their commonest site of origin, produces a purely serous secretion while the other glands produce serous, mucous or mixed secretions, the growths are generally brought together under the convenient, though strictly speaking inaccurate, general designation of " mucous gland tumours ". In any case, the tumours probably arise from the ducts of the glands and the precise nature of the secretion has no influence on their structure or behaviour.

The prognosis for patients suffering from parotid tumours depends upon the method of treatment adopted, and figures quoted for recurrence rates vary widely. Following simple enucleation it is probable that a recurrence rate of 25 to 50 per cent may be expected, though better results are to be obtained from more radical procedures. With regard to tumours of the minor salivary and other mucous glands, precise figures are hard to come by, for the cases are rare and are usually reported in small groups or as single cases, and without waiting to see whether recurrence, which may be long delayed, will take place. Nevertheless, there is a general impression amongst pathologists and clinicians that the prognosis in these cases is worse than in cases of parotid tumour. From a study of the 80 tumours reported here, we hope to decide whether this is in fact the case, and if so, whether it is due to the anatomical sites of the tumours leading to special difficulties in treatment, or to differences in the incidence of histological sub-types. The 80

tumours include all the cases seen at the Middlesex Hospital in the last 30 years, and we have made every effort to trace all the patients. Some patients with mucous gland tumours attend dental hospitals and are treated there ; we have therefore tried to give a more general picture of the site distribution of these tumours by including the cases seen at the Royal Dental Hospital in recent years.

### NORMAL DISTRIBUTION OF MUCOUS GLANDS

As already implied, the term " mucous gland " is used in this investigation to designate the minor salivary and other glands of similar general structure, wherever situated or whatever the nature of their secretion. The distribution of mucous glands relevant to the present study is as follows.

(1) *Oral cavity.*—Glands of minor salivary secretion are plentifully distributed within the oral cavity, and may be conveniently grouped according to situation.

I. *Labial glands.* In the submucosa of the inner surface of the lips.
II. *Retromolar glands.* In the vicinity of the parotid duct opening. They discharge in the third molar region.
III. *Buccal glands.* Situated in the mucous membrane of the cheek.
IV. *Glands of floor of mouth.*
V. *Lesser sublingual glands.* Small glands near the major sublingual gland.
VI. *Glosso-palatine glands.* A group of glands extending posteriorly from the lesser sublingual glands, to ascend in the mucosa of the glosso-palatine fold.
VII. *Palatine glands.* Some 400 glands distributed over the hard and soft palates, and the uvula.
VIII. *Lingual glands.* The anterior lingual glands are situated close to the inferior surface of the tongue, one on each side of the fraenum, near the tip. Other glands are found at the base and borders of the tongue.
IX. *Tonsillar glands.* Many small glands are found outside the capsule of the palatine tonsil. Glands are also present over the pharyngeal tonsil.

(2) *Nose.*—Small glands are found in both the respiratory and the olfactory epithelium.

(3) *Nasal Sinuses.*—Glands are present in all the sinuses. They are smaller and fewer than those in the nasal epithelium.

(4) *Larynx.*—Glands are present in the epithelium throughout the larynx.

(5) *Trachea.*—The lamina propria contains many small glands. In the posterior portion they extend through the muscle layer.

(6) *Bronchi.*—Glands are found as far out in the bronchial tree as the cartilage extends, and usually under the muscle layer.

(7) *Lachrymal Glands.*—These are comprised of one main gland and the tarsal glands on the inner surface of the eyelids.

### PATHOLOGY

The 80 cases are classified on a histological basis as shown in Table I.

TABLE I.—*Site and Histological Type of* 80 *Mucous Gland Tumours*

| Site. | Benign mixed tumour. | Muco-epidermoid tumour. | Basalioma. | Carcinoma. | Totals. |
|---|---|---|---|---|---|
| Palate | 21 | 3 | 5 | 4 | 33 |
| Lip and cheek | 10 | — | 1 | — | 11 |
| Tongue | — | — | 2 | 2 | 4 |
| Peritonsillar | 3 | — | 1 | — | 4 |
| Floor of mouth | — | — | 5 | — | 5 |
| Nose and sinuses | 4 | — | 5 | — | 9 |
| Nasopharynx | 3 | — | 3 | — | 6 |
| Larynx, trachea and bronchi | — | — | 6 | — | 6 |
| Lachrymal gland | — | — | 1 | — | 1 |
| Pyriform fossa | — | — | 1 | — | 1 |
| Total | 41 | 3 | 30 | 6 | 80 |

*Benign mixed tumours*

The term " mixed tumour " is used here in a purely morphological sense, to denote that group of tumours of mucous glands in which epithelial and connective tissue changes are prominent ; it is not meant to convey any notions of histogenesis.

The mixed tumours in the present series were round or ovoid, with smooth, sometimes lobulated surfaces. Most of the tumours appeared to be well encapsulated and circumscribed (Fig. 1), though not infrequently projections from the main body of the growth were noted. Outgrowths of this type have sometimes been misinterpreted as separate or " seedling " nodules of tumour since they appear to form discrete foci of growth lying outside the fibrous tissue capsule (Fig. 2). Foote and Frazell (1953), however, found true multicentric foci in clinically recurrent cases only ; they believe that primary (that is, previously untreated) tumours occur as a single mass in the majority of cases. Patey and Thackray (1953), moreover, have shown by means of serial sections of parotid tumours that apparently discrete nodules are in fact continuous with the main body of the growth, the connecting portion of tissue often being quite a slender strand passing through the capsule. This strand, of course, is readily ruptured when the parent growth is removed by simple enucleation.

Microscopically, the mixed tumours present a characteristically varied appearance. In the major salivary glands some 80 per cent present the familiar picture of strands or sheets of epithelial cells in a chondroid or myxochondroid matrix (Fig. 3). The remaining 20 per cent show variations from what may be called the typical picture, though some features of this are usually present. In the minor salivary and other mucous glands, however, the position is reversed ; it is only the minority of tumours which show extensive areas of chondroid or myxochondroid changes, the vast majority showing such changes in minor degree only.

In these tumours, the stroma may be myxoid or hyaline, while the epithelial elements may be comparatively scanty, taking the form of short strands of spindle-shaped cells (Fig. 4). Or the epithelial cells may be arranged in larger islands, in some cases these being so extensive as to constitute almost a solid epithelial tumour (Fig. 5).

Tubular structures are quite commonly seen. These may be numerous and uniformly distributed throughout the epithelial element of the tumour, and tend to be small and regular. Larger duct-like structures may also be seen ; they are more unevenly distributed and are irregular in size and shape (Fig. 6).

Squamous metaplasia is a not uncommon feature of mixed tumours, varying from small foci of epidermoid cells to fully developed epithelial pearls (Fig. 7). The presence of cells similar to those found in the " onkocytoma " has been reported as occurring in about 10 per cent of mixed tumours (Foote and Frazell, 1953) and also the occurrence of spindle-cells in bundles or in palisade arrangement, suggesting a myoepithelial origin (Sheldon, 1943).

Myxoid, chrondroid, or myxochrondroid changes in the stroma have already been mentioned. Fibrosis and hyalinization may also occur and occasionally fat is present (Fig. 1).

*Malignant mixed tumours*

There is considerable variation in opinion on the incidence of malignant mixed tumours. Ahlbom (1935) had never seen a histologically benign tumour become malignant. Mulligan (1943) reviewed the literature and reported metastases in 12 parotid tumours, 6 sub-maxillary tumours of the palate and 1 of the sublingual gland. However, he considered at least 12 of these tumours to be basaliomas, which are now generally considered as distinct from mixed tumours. On the other hand, none of the 100 mixed tumours in the series reported by Rawson, Howard, Royster and Horn (1950) metastasized. Here the authors had been careful to adopt narrow histological criteria for the diagnosis of mixed tumour. However, Foote and Frazell (1953) also employed strict criteria in the histological diagnosis of benign and malignant mixed tumours and were able to report 57 tumours as malignant mixed out of 877 tumours of all types occurring in the major salivary glands. In the present series there are no examples of malignant mixed tumours.

*Mucoepidermoid tumours*

This group of tumours, considered to be of duct origin, has recently been brought into prominence by the report of Stewart, Foote and Becker (1945) of 45 cases, occurring in some 700 major and minor salivary gland tumours. Earlier accounts of individual cases have been given by Masson and Berger (1924), De and Tribedi (1939) and by others. More recent reports are those of Linell (1948), Rawson, Howard, Royston and Horn (1950), and Smith, Broadbent and Zavaleta (1954).

Histologically, muco-epidermoid tumours are composed of mucus-secreting cells, cells of epidermoid type and an intermediate variety of cell, in varying proportions. In the tumours of a lower grade of malignancy cysts are frequent, these being lined by mucous cells or by non-mucous columnar or epidermoid cells (Fig. 8). Mucus is often produced abundantly, resulting in rupture of the cystic spaces and escape of the secretion into the stroma, in which a foreign body reaction may then develop. In tumours of a higher grade of malignancy epidermoid and intermediate cells predominate and cyst formation is infrequent.

Rawson and his co-workers found 11 muco-epidermoid tumours in their series of 160 tumours of the major and minor salivary glands, 8 being of low and 3 of high malignancy. All the tumours of low grade malignancy were from the parotid ; the remaining 3 were derived from the tongue, the parotid and the submaxillary glands. Smith, Broadbent and Zavaleta (1954) found 7 muco-epidermoid tumours in their series of 32 oral mucous gland tumours, an incidence of 30 per cent compared with the 5 per cent incidence in Stewart, Foote and Becker's (1945) series, the 7 per cent of Rawson *et al.* (1950) and the 11 per cent of Foote and Frazell (1953). The 3 muco-epidermoid tumours in the present series give an incidence of just under 4 per cent. It has already been pointed out that benign mixed tumours of the minor salivary and mucous glands tend towards a ductular form, together with abundant epidermoid features; it is possible that tumours of that type may sometimes be considered as muco-epidermoid.

*Basalioma*

Tumours are encountered occasionally in the major salivary glands and relatively more frequently in the mucous glands of the upper respiratory tract, which have a very characteristic and easily recognisable histological appearance but which suffer from a large number of aliases. One of the earliest recorded examples of a tumour of this type which had invaded the orbit, having apparently arisen in the nasal sinuses, was published by Billroth (1859) under the name of "cylindroma". He gave it this name because the cylindrical masses of epithelial cells were surrounded by prominent hyaline or glassy sheaths staining a uniform pale pink with eosin. Tumours with this histological characteristic are found arising from a variety of glands, ard it is to the type arising in sweat glands that the name is most commonly applied at the present time. Unfortunately, since Billroth's original description, the term "cylindromatous" has often been used with a meaning quite different from his and it is now regarded by many writers as a term to be avoided in view of its frequent misinterpretation. Krompecher (1908) included tumours of this type as intranasal basal cell carcinomas, calling them "cylindromatous basalioma". In the monograph on tumours of the nose by Ringertz (1938) the term "basalioma" is used, an example which we shall follow. The term most often used in America for these growths is "adenoid cystic carcinoma" which, however, does not draw attention to their undoubted affinities with growths of the basal cell group.

Histologically, the basalioma consists chiefly of small deeply staining cells, though sometimes rather larger cells may preponderate. A wide variety of patterns may be seen, often in the same tumour. Figs. 9 and 10, for example, are illustrations of fields from the same specimen. In Fig. 9 the tumour is growing in solid masses, some showing central necrosis. To the left of Fig. 10 the cell clumps are much smaller, and tend to form acini containing mucoid material, while towards the right acinar formation is not marked, the cells forming strands and columns in a hyaline stroma. Frequently the stroma becomes mucoid, the epithelial cells persisting in columns, but occasionally so much mucoid change takes place that only the cell groups at the periphery remain. An extreme example is seen in Fig. 11, where the appearances might easily suggest benign mixed tumour.

Mucoid changes also occur in such a manner that the cell groups are broken up into very fine strands (Fig. 12). Here again the appearances simulate those found in some benign mixed tumours. In Fig. 13 mucoid change has proceeded in such a way as to produce quite large cystic spaces in the stroma, and in Fig. 14, from another tumour, well-marked cribriform arrangement of the cells is evident.

In some cases transitions to a more obviously malignant histological picture are seen (Fig. 15, which comes from the same case as Figs. 9 and 10), but even when the tumour remains apparently well differentiated there may still be infiltration of surrounding tissues. There is a special tendency to invasion of the perineural lymphatics (Fig. 16), whilst the haversian systems of bone may be involved without alteration of the general structure (Fig. 17).

*Carcinoma*

Apart from those carcinomas which have arisen in benign mixed tumours, and those which conform to the basalioma type, there still remain indeterminate tumours, variously described as of solid type, mucous cell or acinic cell types, or anaplastic. Such tumours are relatively infrequent ; they are described briefly and figured by Foote and Frazell (1953).

In the present series there are 6 carcinomas, 4 of the palate and 2 of the tongue. In the previous section it was noted that in some basaliomas there were transitions to a more frankly malignant arrangement. If such areas were to be viewed out of their context they could well be considered simply as undifferentiated carcinoma. In one of the carcinomas in this series there were areas which strongly suggested a basiliomatous origin, while in the others only small amounts of material were available for study.

### CLINICAL FEATURES

### *Palate*

Of the 80 tumours reviewed in this paper no less than 33 (41 per cent) occurred in the roof of the mouth. The majority of these arose near the junction of the hard with the soft palate, but a few developed in the alveolus. There are no mucous glands in the anterior portion of the hard palate and no tumours were attached in that situation, although some were so large that they were in contact with most of the roof of the mouth.

*Histology*

On histological examination the numbers of cases in each group were as follows :

| | |
|---|---|
| Mixed type . . . . . . . | 21 (64 per cent). |
| Basalioma . . . . . . . . | 5 (15 per cent). |
| Carcinoma . . . . . . . . | 4 (12 per cent). |
| Mucoepidermoid . . . . . . | 3 ( 9 per cent). |

*Age and sex and length of history*

Of the 33 patients there were 16 men and 17 women, but all four with carcinomata were men.

The youngest patient was 26 years of age and the oldest 80 when they first attended for treatment, but the latter had had her tumour for over 37 years. In about half the cases the tumour was first noticed before the age of 30 and in 3 cases (all of the mixed type) before the age of 20.

Slowly growing tumours of this kind, changing little from year to year, sometimes reach a large size before the patient sees fit to ask for advice, and in over 25

per cent of these cases there was a definite history of a tumour for more than 10 years and in several others a history of " many years ".

*Size*

In the majority of cases at the time the patients presented for examination the tumour was from 2–4 cm. in diameter (Fig. 18), although some of the tumours which had been present a long time were very much larger. The largest of all occurred in a man of 33 in whom the tumour had first appeared at the age of 13. It filled almost the whole of the mouth and extended in addition well up into the nasopharynx above and rested on the epiglottis below (Fig. 19). It is not surprising that he had dysphagia and some dyspnoea on bending forwards. After removal the specimen measured nearly 4 inches across (Fig. 20).

*Symptoms*

Most of the patients reported to their doctors because of the presence of a painless swelling in the palate, but some reported to their dentists because a denture ceased to fit, and in a few the tumour was discovered accidentally during dental treatment for some unrelated condition. Occasionally the surface of the tumour had become ulcerated with some associated bleeding.

*Treatment and results*

The methods of treatment adopted may be classified under four main headings: local excision (enucleation), wide excision, diathermy excision and radiotherapy. Follow-up of results to the present time or to death has been possible in all except 2 cases, but several cases have been treated in the last few years and in 8 cases the follow-up period has been less than 5 years. Even 5 years must be regarded as valueless in following up this type of case because only one recurrence of a mixed tumour has been noticed in less than 7 years and recurrences have occurred after periods of up to 17 years. It seems most useful to consider the forms of treatment employed and the results obtained in relation to the histological groups.

*Mixed tumours*

In 8 cases in this histological category, the primary form of treatment was simple local excision and in 4 of these cases there has been recurrence; in one case after 2 years, in others after 7 and 17 years, and in the fourth after 14 years (and again after a further period of 16 years). Of the 4 cases in which there has been no recurrence so far, 1 has been followed up for only one year and the others have been observed for 12, 20 and 22 years. Thus there has been recurrence in 50 per cent of the mixed tumours of the palate treated by enucleation alone, and if the case followed for only a year were to be excluded from the survey, the recurrence rate would be 57 per cent.

One tumour was treated by radiotherapy alone and this recurred rapidly and again recurred after a further course. The patient then returned to his native country and cannot now be traced.

There has been no recurrence to date in patients treated by wide excision (5 cases, of which one had preliminary deep X-ray therapy), diathermy excision

(5 cases, of which one had preliminary deep X-ray therapy) or radiotherapy followed by local excision (1 case), but no definite conclusions can be drawn from this as in only 2 of these cases has the follow-up period been over 10 years.

In the remaining case, treated in 1926, adequate details are not available to classify the form of treatment.

*Basaliomas.*—Of the five patients in this group one received no treatment for the tumour in question because of another independent tumour—a squamous cell carcinoma—from which he died 3 years later.

One patient had the tumour widely excised four years ago and is now well and apparently free from recurrence.

The remaining three patients have all had extensive treatment on several occasions, with many recurrences. One patient died 5 years after coming under

---

EXPLANATION OF PLATES.

FIG. 1.—Palatal mucous gland tumour of mixed type, showing a well-marked fibrous capsule separating the growth from a normal palatal gland at the upper left of the figure. × 40.

FIG. 2.—Benign mixed tumour of lip showing an extension of the main growth outside the capsule. The plane of sectioning is such that the appearance of a separate nodule is given. × 35.

FIG. 3.—Typical mixed tumour of peritonsillar region, showing cartilage-like areas in the stroma. × 100.

FIG. 4.—Hyaline change in the stroma of a mixed tumour of the palate compressing the tumour cells. × 135.

FIG. 5.—Another field from the tumour shown in Fig. 4 to illustrate the solid nature of the growth in this area. × 65.

FIG. 6.—A mixed tumour of the palate showing the formation of duct-like structures. × 35.

FIG. 7.—Areas of squamous differentiation with cysts filled with keratin in a mixed tumour of the palate. × 35.

FIG. 8.—Mucoepidermoid tumour of the palate. Two fields showing a mucoid cyst and a clump of squamous cells. × 120.

FIG. 9.—Basalioma of floor of mouth. Tumour cells in solid masses, the largest of which shows central necrosis. × 55.

FIG. 10.—Another field from the tumour illustrated in Fig. 9, showing transition from acini to strands. × 55.

FIG. 11.—Basalioma from floor of mouth showing mucoid change in the stroma; this may be sufficiently extensive to lead to confusionn with mixed tumour. × 90.

FIG. 12.—Basalioma of floor of mouth. Mucoid change has led to breaking up of the epithelial cell groups. × 110.

FIG. 13.—Basalioma of antrum showing cribriform pattern. × 50.

FIG. 14.—Basalioma of nasopharynx. Another example of the cribriform type. × 80.

FIG. 15.—Another field of the tumour illustrated in Fig. 4 and 5, showing undifferentiated growth with invasion of a small vessel. × 55.

FIG. 16.—Invasion of perineural lymphatics in the neighbourhood of the Gasserian ganglion by a basalioma from the nasopharynx. × 30.

FIG. 17.—Basalioma of palate invading bone. × 30.

FIG. 18.—A mixed tumour of the palate which had been growing for 30 years.

FIG. 19.—Large mixed tumour of the palate. Radiograph showing the lower pole of the growth resting on the epiglottis.

FIG. 20.—Specimen removed from the patient whose radiograph is shown in Fig. 19.

FIG. 21.—Mixed tumour of lip. One of many serial sections which showed that the multiple foci of recurrent growth were discrete. Contrast with Fig. 2. × 4.

FIG. 22.—Bisected operation specimen showing a diffusely nodular basalioma in the subglottic region.

1

2

3

4

5

6

7

8a

8b

9

10

11

12

Ranger, Thackray and Lucas.

13

14

15

16

17

21

18

19

20

22

observation and had active tumour present at the time of death in spite of local excision, subsequent wide excision and two courses of deep X-ray therapy. Another patient had a wide excision followed by a course of deep X-ray therapy (6000r) but now, 7 years later, has an extensive local recurrence (first noted 2 years ago) and also multiple pulmonary metastases. The other patient had a diathermy excision with a recurrence 3 years later, for which a wide excision was done. However, after another 5 years there was further recurrence and this was treated by further diathermy excision followed by deep X-ray therapy. Within a year there was another recurrence and this was treated by local excision and radon seed implantation. Further recurrence has taken place and now, 11 years after first coming under observation, she still has active tumour present.

*Carcinoma.*—Four patients had their tumours diagnosed as malignant in the first biopsy. Of these, two died within a few years with active tumour and two are alive and apparently free of tumour after periods of 1 and 10 years.

Of the two who have died one was treated by a wide excision and this was followed by a further excision when the tumour recurred. He died 3 years after coming under observation with tumour still present. The other patient was treated initially by teleradium and then by local implantation of radium for a recurrence, but he died with metastases within 2 years of starting treatment.

The two who are still alive have both had recurrences. The first had deep X-ray therapy and then wide excision for neoplasm which was still present 9 months after the radiotherapy. There is no recurrence evident now, but only 6 months have elapsed since the excision. The other patient had deep X-ray therapy and then 2 years later had diathermy excision for a recurrence. He has had no further local recurrence but has had a bilateral block dissection of the neck for glands which were heavily invaded histologically. He is now well and apparently free from tumour 10 years after the initial treatment and 3 years after the last block dissection.

*Mucoepidermoid tumours.*—There were three examples of this type of growth ; two are recent, the other has repeatedly recurred over a period of 16 years during which time there has been much destruction of bone.

One of the recent cases, a woman of 46 years, is of interest. The patient complained of a swelling on the palate which, being fluctuant on examination, was diagnosed as a mucous cyst or abscess. On being opened a thick purulent fluid escaped, thus seemingly confirming the clinical diagnosis. Histological examination of the " abscess " wall, however, showed mucoepidermoid tumour.

### *Lip and cheek*

There were 11 tumours in this group, 1 in the cheek and the remainder in the lip. All 10 of the lip tumours were in the upper lip and none in the lower, which seems a remarkable coincidence. Several of the patients gave long histories of the presence of the lump, the longest being 20 years, by which time it was an inch and a quarter in diameter. This was the largest in the group. Histologically ten of the tumours were of the benign mixed type and one was a basalioma. Most of the tumours were removed in the Casualty Department and it has not been possible to trace the patients for news of their further progress. The tissues of the lip are soft and it was obvious from the histological sections that most of the tumours had been removed with some surrounding tissue ; it is only in firmer sites that there is a temptation to shell them out from the surrounding fibrous zone. Recurrent cases of such lip tumours are very rarely encountered but there was one in this series. A man had a mucous gland tumour of his lip removed at the age of 41. It recurred and was re-excised 5 years later. It recurred again and was re-excised 3 years later and again 5 years later when it was described as having been widely excised. After another 6 years there was a further recurrence, section of which shows the characteristic feature that these recurrences are multiple (Fig. 21). Thus this patient with a mixed tumour has had four recurrences in the course of 19 years.

### *Tongue*

There were no mixed tumours among the 4 tumours of the tongue.

Two were diagnosed as of mucous gland origin but highly suggestive of malignancy. Both patients were treated by irradiation but died of other causes before the outcome could be assessed ; in one at least there seemed to have been a favourable response. Two tumours were basaliomas.

A basalioma of the tongue in a man of 65 is of importance. He complained of pain in the right side of the tongue and on examination a hard, ill-defined swelling was found in the right side of the posterior third of the tongue, apparently invading the adjoining alveolus. There were no glands and the Wassermann reaction was negative. On the assumption that this was a squamous cell carcinoma an extensive operation was performed, involving removal of the ramus and half an inch of the body of the mandible and diathermy excision. The specimen was described as a portion of tongue 2 inches in diameter covered with mucous membrane, from the centre of which projected a pale, nodular growth 1 inch in diameter. On section, the underlying muscle was found to be infiltrated with pale growth. This man died 15 years later at the age of 80, of acute bronchitis, free from any recurrence of his tumour.

The other basalioma was treated by teleradium 4 years ago and there is no sign of recurrence.

### *Peritonsillar region*

There were 4 tumours in the series which arose in the peritonsillar region. When studying microscopical sections of tonsils which have been dissected out it is not uncommon to see mucous glands in the tissue deep to the main body of the tonsil, their ducts either skirting the gland or, rarely, apparently entering it and discharging into a crypt. Anatomically, of course, the problem may arise whether a given tumour arises in fact from the peritonsillar mucous glands or in the deep part of the parotid gland, since tumours in the latter situation sometimes have pharyngeal extensions.

Three of the 4 tumours, in women of 35 and 40 and in a man of 53, were of the benign mixed type. The fourth was a basalioma.

The man of 53 with the mixed tumour had had a swelling of the right side of his throat for 6 months, for which he had been given a course of X-ray therapy with little apparent effect. On examination he was found to have a firm swelling the size of a small egg behind the tonsil, which was pushed forward and inwards. Biopsy showed this to be a typical mixed tumour and it was excised from inside the mouth after removal of the tonsil, being noted as very hard, not very vascular and with a good capsule. The histological section showed the tumour to be little affected, apparently, by the previous irradiation (Fig. 2). This patient remained well until he died of coronary disease 11 years after the operation.

The one case of basalioma in this group was a man of 45 who had complained of nose bleeding and left earache for a year. A left supratonsillar growth involving the soft palate was found. He was treated by deep X-rays and interstitial radium, with some reduction in size of the swelling. Two years later it was again enlarging and was excised by diathermy. He died a few months later as a result of haemorrhage from this site.

### *Floor of Mouth*

All 5 tumours occurring in the floor of the mouth were of the basalioma type. Two patients have been treated within the last 3 years, one by wide excision and the other by teleradium and radium implant, and these two are the only ones alive. The remaining three were treated in the first instance by local excision, but they all had recurrences. These patients have since died—two from further recurrences and one post-operatively after a second and much more radical excision.

### *Nose and Sinuses*

The 9 tumours of mucous gland origin in the nose and sinuses, 4 mixed tumours and 5 basaliomas, were situated as follows: 2 in the ethmoid region, 2 in the antrum and 1 each arising from the frontal sinus, floor of nose and inferior turbinate. The remaining two were so large that their precise site of origin could not be determined.

One of the mixed tumours was small and was discovered by accident. The patient complained of bleeding from the left nostril; on examination a tumour the size of a bean was found growing from the right inferior turbinate. It was removed and all should be well. Another mixed tumour, the size of a cherry, growing from the floor of the nose was removed surgically, but has recurred four years later. A third patient who complained of a stuffy nose on the right side was found to have a polypoid, friable mass filling the right nasal cavity; this was removed and has not recurred in 15 years. The fourth mixed tumour of the nose presented with epistaxis and loss of sight in the left eye. X-ray examination showed an extensive tumour with destruction of the ethmoid cell walls and of the margins of the left optic foramen. Biopsy showed an extensively pseudo-cartilaginous mixed tumour with considerable areas of necrosis. The growth was clearly beyond the reach of surgery; the patient was given deep X-ray therapy but died from local haemorrhage and infection a few months later.

The 5 basaliomas have run a more morbid course than the mixed tumours, since two of the patients have already died whilst the remaining three have recurrences.

A woman of 58 noticed tears running down her right cheek. Her nasolachrymal duct was dilated. Soon afterwards a swelling appeared on her forehead; as it enlarged she noticed dimness of vision in one eye with intermittent epistaxis. On examination there was a firm, smooth, rubbery swelling of her forehead in the midline and an ulcerated swelling also presented in the nose, a biopsy of which showed typical basalioma.

Radiotherapy produced some temporary improvement, but she was dead in 3 years from the local effects of this invasive tumour of the frontal sinus.

A young man of 22, a hospital porter, had noticed left nasal obstruction for a year with prominence of the left eye. X-ray examination showed opacity of the left antrum with thinning of its walls, and a biopsy proved the antrum to be the site of a basaliomatous mucous gland tumour. He had radium treatment followed by a palatal antrostomy which revealed very extensive growth. The tumour, which was temporarily controlled by the radium treatment, recurred 2 years later, when he had a course of deep X-ray therapy. This led to considerable necrosis of the tumour, so that a large cavity representing antrum, orbit and nasal cavity opened on to the front of his face. The eye had ceased to function after the initial radium treatment and at this stage necrosed and was lost. A year later he had a further recrudescence, again treated by deep X-ray therapy, but by now there was direct invasion of the brain by tumour and he died 5 years from the onset of his illness.

The three other cases are still under treatment. A man of 61 noticed pricking and numbness of his left cheek 2 years ago, followed by left-sided headaches, epistaxis and pain in the left eye with decreasing vision and proptosis. X-ray examination showed opacity of ethmoids, antrum and sphenoid on the left with erosion of bone, and a biopsy showed basaliomatous mucous gland tumour. He had deep X-ray therapy to the extent of 6000r, followed later by exenteration of the orbit, ethmoids and sphenoid. Tumour was still present and apparently viable in the left sphenoid region but not elsewhere. Now, a year later, there is obvious recurrence in the wall of the cavity.

Another middle-aged man with a tumour of the antrum is of interest in that the tumour in his nose has been kept under control by irradiation, though with recurrence from time to time, but 3 years ago he was found to have lung secondaries.

*Nasopharynx*

There were five women, four of them in their forties, and one man of 37 in this group. Histologically 3 of the tumours proved to be of the mixed type and 3 were basaliomas.

Three patients with nasopharyngeal tumours of the mixed type all presented with obvious swellings, which were removed without great difficulty. The course of events in the three patients with basaliomas of the nasopharynx was far different and will be illustrated by some notes about one of them.

A woman of 44 years of age first suffered from severe right-sided headaches in 1948. A year later she suddenly became deaf in the right ear, and a year after this noticed blurring of vision in the right eye. The eye condition was treated as glaucoma and was said to be " not typical ". The next year she began to suffer from attacks of epistaxis with pain in the right side of the face, and yet a year later was found to have loss of the whole right temporal field which progressed to complete blindness. She was referred to a neuro-surgeon who exposed the right middle cranial fossa and found " firm, flat, pink, extradural granular tissue, involving the bone. It had destroyed foramina ovale et rotundum and was infiltrating the 2nd and 3rd divisions of the trigeminal nerve " (Fig. 16). The mucous gland tumour, of which this was clearly an extension, was identified in biopsies from several sites within the nose and an X-ray examination at this time showed an opaque antrum with partial destruction of lateral and medial walls, opaque posterior ethmoid and some destruction of the medial part of the greater wing of the sphenoid. She then received deep X-ray therapy and after 6000r was much improved, though a palatal antrostomy a year later still showed viable tumour. After a further course of irradiation her antrum had cleared, hearing in the right ear had returned in a distorted form and she gained a stone in weight. Now, a year later, there is no obvious recurrence.

*Larynx, Trachea and Bronchi*

Of the 6 tumours in this group, one was situated in the larynx below the cord, three were in the trachea and two in the main bronchi.

Histologically all these tumours were typical basaliomas. These growths in the bronchi have been in the past merged into the general heading of bronchial adenoma.

The two patients with bronchial tumours, one man and one woman both in their forties, are still alive. The man had suffered attacks of " congestion of the lungs " each winter since 1940, and when he attended hospital in 1948 complained of increasing dyspnoea between the attacks. Bronchoscopy showed multiple polypoid nodules in the trachea and left bronchus. Biopsy showed these to be mucous gland tumours and he was treated with deep X-ray therapy followed by removal of as much tumour as possible through a bronchoscope. This resulted in great improvement in his condition, but when he was bronchoscoped again 2 years later recurrent tumour was removed, and similarly again 3 years later. He is still alive, 6 years after the first treatment, with varying severity of dyspnoea and cough.

The woman with a bronchial basalioma is also still living ; she first complained of a troublesome but non-productive cough in 1946. Two years later she was bronchoscoped and a tumour in the left main bronchus was partially removed. By 1951 she still had a productive cough and dyspnoea. On bronchoscopy there was seen a sessile tumour almost occluding the trachea, arising from the left main bronchus. She was treated by deep X-ray therapy, 5000r, and her condition was much improved. At bronchoscopy afterwards there was an adequate airway. A year later, although the notes record her as being very well, there was no air entry into the left lung.

A man of 53 complained of attacks of dyspnoea for which he had been treated as an asthmatic for a year. He was found to have a tumour of the trachea for which he had more than one course of deep X-ray therapy and radon seed implantation, biopsies at intervals showing tumour to be still present. He continued with attacks of dyspnoea and was admitted to hospital again 5 years after the first diagnosis, the dyspnoea having become urgent. A Souttar's tube was inserted, and tracheotomy performed. He died shortly after of bilateral broncho-pneumonia with purulent pleural effusion. A description of the local condition at post mortem was as follows : " From the 4th to the 12th tracheal ring in the left antero-lateral and lateral wall there is a hard, necrotic, malignant ulcer. The lumen of the trachea is not obstructed. On section through the tracheal wall several rings in the middle of the growth have been destroyed and several necrotic rings are protruding into the lumen. The growth has extended through the tracheal wall on the left and for most of its length is $\frac{1}{2}$ to $\frac{3}{4}$ inch thick. There are no distant metastases."

The other two patients, whose tumours were noted as being in the trachea 2

inches below the cords, both came to hospital complaining of increasing dyspnoea, cough and stridor for a year or so. The woman, who was in her early thirties, was bronchoscoped and found to have her trachea almost blocked by growth. Biopsy showing this to be a mucous gland tumour, she first had radium on a Tudor Edwards applicator for 48 hours, 4500r. A bronchoscopy 2 months later showing residual growth, she was treated by deep X-ray therapy. Three months later another bronchoscopy showed tumour still present, so early the next year a tracheectomy was performed. She died of haemoptysis 2 months later. At post mortem the greater part of the trachea was seen to have been replaced by a spiral steel tube which was well embedded, but the airways were full of blood which appeared to have come from the junction of the lower end of the steel spiral to the stump of the trachea, at which site tumour was still present. There were no secondaries.

The man had a very similar story. After attempts at destroying the tumour by radiotherapy had failed, a total tracheectomy with plastic reconstruction was performed. He died a week after the operation with bilateral bronchopneumonia and mediastinal cellulitis, the spiral steel artificial trachea covered by fascia from the thigh lying in a long abscess cavity. The lines of junction above and below appeared to be intact. Although the trachea had been removed from a point two rings above the carina to one ring below the cricoid there was still tumour at the lower anastomosis.

The laryngeal tumour was in a young woman who had complained of a husky voice for 6 months with dyspnoea on exertion. On examination the cords were normal, but there was a subglottic mass. Direct laryngoscopy showed tumour almost encircling the air passage 2 cm. below the vocal cords and extending over $1\frac{1}{2}$ cm. Total laryngectomy was performed (Fig. 22). Histological examination showed well differentiated basalioma infiltrating the entire laryngeal wall and reaching the external surface of the specimen.

### *Lachrymal Gland*

There was only 1 tumour in the series which arose in the lachrymal gland. This basalioma was in a man of 34 years, the initial treatment being local excision and radium implantation. A recurrence 4 years later was treated by wide excision, but a further recurrence occurred the following year. In spite of more radium he died 6 years after diagnosis with secondaries in the frontal lobes of his brain.

### DISCUSSION AND CONCLUSIONS

The findings in these 80 cases clearly confirm the suspicion that the prognosis in tumours of the minor salivary and the mucous glands is worse than in those of the major salivary glands, since at the time of writing half of them had already recurred or persisted in spite of treatment, though quite a number have been followed for only one or two years as yet. In view of the considerable delay in the appearance of the recurrent tumour in some cases this figure of 50 per cent is certainly an underestimate.

It was hoped to decide whether this worse prognosis was due to the anatomical sites of the tumours increasing the difficulty of diagnosis and treatment, or to a preponderance of more sinister histological types of tumour. It appears that both factors play a part. In the lip, for example, where all but one of the tumours were of the mixed type and where surgery is simple, the prognosis is excellent whereas with similar tumours in the palate the recurrence rate is higher than in tumours of the parotid gland. Of equal or greater importance than anatomical site in determining the poor prognosis of this group as a whole is the fact that the ominous basaliomas formed 35 per cent of the total as against about 2 per cent of all parotid tumours. In the case of the larynx, trachea and bronchi, not only is surgical removal much more difficult than in the lip, but all the tumours were of the basaliomatous type. It has been stressed that a feature of this type of growth is its infiltrative habit, and in all the operation specimens the tumours of the larynx or trachea had extended to the outer surface of the tissue removed. Had the more circumscribed mixed type of growth been encountered in this situation some of the technically successful operations performed might have resulted in cure.

A few general conclusions on the treatment of these growths can be drawn from a consideration of this series of cases. Simple enucleation of tumours of the mixed type is very likely to be followed by recurrence, though possibly long delayed; they should be excised with a margin of normal tissue. Radiotherapy, where employed, appears to have had little or no effect on tumours of the mixed type. The basaliomatous growths form a less definite tumour mass and there is nearly always microscopic infiltration beyond their macroscopic limits, so that wide removal in the first instance appears to hold out the only hope of cure. Once recurrence has taken place the chance of reaching beyond the infiltrative margin at a second operation appears slender. Earlier writers have been well aware of the behaviour of these basaliomatous growths, but in stressing their malignant infiltrative properties have not laid sufficient emphasis on the possibility of their extirpation by a sufficiently radical operation in the first instance. The satisfactory or even dramatic response of these tumours to irradiation has also been well recognised in the past, though they have been recorded as almost invariably recurring after such treatment. One of the aims of this investigation was to see whether modern and improved radiotherapeutic techniques could permanently cure these basaliomatous growths. Unfortunately, tumours of this type treated in the last year to two have already recurred. Nevertheless, worth while palliation can be achieved, and where further surgery is out of the question radiotherapy is certainly indicated. The difference in response to irradiation of the mixed and basaliomatous types of mucous gland tumour is very real and is a strong reason for the histological subdivision of mucous gland tumours. As already implied, a number of tumours will be encountered in which examination of several areas may be necessary before it becomes apparent into which group the tumour should be placed.

Few cases of frank carcinoma of mucous gland origin have been encountered in the series, but in some of them the grade of malignancy has been low and years of freedom have followed removal of the primary growth and block dissection of invaded glands of the neck.

### SUMMARY

The clinical course and histological features of 80 tumours of mucous gland origin are described. Attention is drawn to the varying incidence of histological types in the different anatomical sites considered, and the results of various forms of treatment are compared.

ACKNOWLEDGMENTS

Our thanks are due to the surgeons in charge of these cases for permission to refer to the clinical records. Part of the expenses of this investigation was defrayed by the British Empire Cancer Campaign.

### REFERENCES

Ahlbom, H. E.—(1935) *Acta Radiol.*, Suppl. **23**.
Billroth, T.—(1859) *Virchows Arch.*, **17**, 357.
De, J. N. and Tribedi, B. P.—(1939) *J. Path. Bact.*, **49**, 432.
Foote, F. W. and Frazell, E. L.—(1953) *Cancer*, **6**, 1065.
Krompecher, E.—(1908) *Beitr. f. path. Anat.*, **44**, 51, 58.
Linell, F.—(1948) *Acta Path. Microbiol. Scand.*, **25**, 801.
Masson, P. and Berger, L.—(1924) *Bull. Assoc. Franc. p. L'ètude du Cancer*, **13**, 366.
Mulligan, R. H.—(1943) *Arch. Path.*, **35**, 357.
Patey, D. H. and Thackray, A. C.—(1953) *Ann. Rep. Brit. Emp. Cancer Campgn.*, **31**, 74.
Rawson, A. J., Howard, J. H., Royster, H. P., and Horn, R. C.—(1950) *Cancer*, **3**, 445.
Ringertz, N.—(1938) *Acta Oto-laryngol., Suppl.* **27**,
Sheldon, W. H.—(1943) *Arch. Path.*, **35**, 1.
Smith, A. G., Broadbent, T. R. and Zavaleta, A. A.—(1954) *Cancer*, **7**, 224.
Stewart, F. W., Foote, F. W. and Becker, W. R.—(1945) *Ann. Surg.*, **122**, 820.